# The contribution of major depression to the global burden of ischemic heart disease: a comparative risk assessment

**DOI:** 10.1186/1741-7015-11-250

**Published:** 2013-11-26

**Authors:** Fiona J Charlson, Andrew E Moran, Greg Freedman, Rosana E Norman, Nicolas JC Stapelberg, Amanda J Baxter, Theo Vos, Harvey A Whiteford

**Affiliations:** 1 Queensland Centre for Mental Health Research, Brisbane, Australia; 2 School of Population Health, University of Queensland, Brisbane, Australia; 3 Division of General Medicine, Department of Medicine, Columbia University Medical Center, New York, USA; 4 Institute for Health Metrics and Evaluation, University of Washington, Seattle, USA; 5 Queensland Children’s Medical Research Institute, University of Queensland, Brisbane, Australia; 6 School of Psychology, Griffith University, Brisbane, Australia

**Keywords:** Depression, Ischemic heart disease, Comparative risk assessment, Burden of disease

## Abstract

**Background:**

Cardiovascular disease and mental health both hold enormous public health importance, both ranking highly in results of the recent Global Burden of Disease Study 2010 (GBD 2010). For the first time, the GBD 2010 has systematically and quantitatively assessed major depression as an independent risk factor for the development of ischemic heart disease (IHD) using comparative risk assessment methodology.

**Methods:**

A pooled relative risk (RR) was calculated from studies identified through a systematic review with strict inclusion criteria designed to provide evidence of independent risk factor status. Accepted case definitions of depression include diagnosis by a clinician or by non-clinician raters adhering to *Diagnostic and Statistical Manual of Mental Disorders* (DSM) or *International Classification of Diseases* (ICD) classifications. We therefore refer to the exposure in this paper as major depression as opposed to the DSM-IV category of major depressive disorder (MDD). The population attributable fraction (PAF) was calculated using the pooled RR estimate. Attributable burden was calculated by multiplying the PAF by the underlying burden of IHD estimated as part of GBD 2010.

**Results:**

The pooled relative risk of developing IHD in those with major depression was 1.56 (95% CI 1.30 to 1.87). Globally there were almost 4 million estimated IHD disability-adjusted life years (DALYs), which can be attributed to major depression in 2010; 3.5 million years of life lost and 250,000 years of life lived with a disability. These findings highlight a previously underestimated mortality component of the burden of major depression. As a proportion of overall IHD burden, 2.95% (95% CI 1.48 to 4.46%) of IHD DALYs were estimated to be attributable to MDD in 2010. Eastern Europe and North Africa/Middle East demonstrate the highest proportion with Asia Pacific, high income representing the lowest.

**Conclusions:**

The present work comprises the most robust systematic review of its kind to date. The key finding that major depression may be responsible for approximately 3% of global IHD DALYs warrants assessment for depression in patients at high risk of developing IHD or at risk of a repeat IHD event.

## Background

Cardiovascular disease and mental health both hold enormous public health importance. Globally, they have both ranked highly in terms of burden of disease and prevalence [1]. In the most recent Global Burden of Disease 2010 Study (GBD 2010) ischemic heart disease (IHD) and stroke rank first and third, respectively, in terms of disability-adjusted life years (DALYs), while major depressive disorder (MDD) ranked 11th out of 291 disease and injuries [2]. These disorders have all increased in ranking positions since 1990 estimates. When considering the morbidity component alone, 5 mental disorders (MDD, anxiety, bipolar disorder, schizophrenia and dysthymia) feature in the top 20 causes of years lived with disability (YLD).

Although disorder-specific GBD 2010 estimates can be used to guide health policy and planning, estimates of the additional burden due to disorders that are risk factors provide an additional evidence base for developing preventative health policy. The Comparative Risk Assessment (CRA) component of the GBD 2010 is a systematic and quantitative assessment of changes in population health that would result from modifying the population distribution of exposure to a risk factor or a group of risk factors [3,4]. The GBD 2010 is the first to include mental disorders as independent risk factors for other health outcomes globally. Quantifying the proportion of health outcomes attributable to mental disorders has thus far been neglected.

The association between major depression and IHD is well documented [5-14]; however, previously applied review methodologies have not robustly tested the temporal relationship between major depression and IHD, which is essential for clarifying the potential role of depression as an independent risk factor for IHD given the well-established bidirectional relationship between these two diseases. There is increasing evidence for considering depression as an independent risk factor in the development of IHD. A detailed rationale for examining major depression as an independent risk factor for the development of IHD, rather than an association, has been discussed elsewhere [15]. Temporal and dose–response relationships have been proposed in the literature, as well as plausible behavioral and biological pathways. However the possibility remains that depression could be a non-causal risk marker in IHD [16].

This study aims to systematically and quantitatively assess major depression as an independent risk factor for the development of IHD. We present findings from a systematic review of the literature and estimate the overall risk of developing IHD in those suffering from major depression, estimate number of cases of IHD in the population which may be caused by major depression, and report the disease burden of IHD attributable to major depression. These calculations are presented for 21 world regions, both sexes, 11 age groups, and for 1990, 2005 and 2010 time periods.

## Methods

### Case definitions

According to *Diagnostic and Statistical Manual of Mental Disorders*, fourth Edition (DSM-IV) criteria, MDD is characterized by one or more major depressive episodes lasting for at least 2 weeks [17,18]. There is acknowledgement in the literature that the heterogeneity of depression has implications for research [19]. We reduced this heterogeneity by requiring a case definition of MDD in the studies analyzed to be diagnosis by a clinician or by non-clinician raters adhering to DSM or *International Classification of Diseases* (ICD) diagnostic classifications [20]. For studies that used symptom scales, we required that these map to DSM/ICD diagnostic thresholds as determined by consensus of three of the authors. As we accept measures that are not strictly diagnostic, we will refer to the exposure in this paper as major depression (that is, presence of moderate to severe depressive symptoms) as opposed to the DSM-IV category of MDD.

Similarly, we draw upon the ICD coding scheme for our case definition of IHD. In line with the basic GBD definitions of IHD we accepted cases of ICD-9 codes 410 to 414 or ICD-10 codes I20 to I25 [21]. Angina pectoris alone was not considered an acceptable proxy for IHD due to its subjective nature and usual measurement by self-report alone. However, it was expected that angina (ICD-9 413 and ICD-10 120) would be included in the IHD case definition of many studies. Study estimates that included cases determined by self-report, physician panel review of medical records, or ICD codes other than previously mentioned were also taken into consideration and accepted after discussion and consensus with other investigators. Myocardial infarction (MI) was considered to be an acceptable proxy for IHD and the World Health Organization (WHO) 2007 criteria for MI were accepted [22]. Consistent with the GBD 2010 definition of IHD, asymptomatic (‘silent’) IHD captured by electrocardiogram or cardiac imaging were not included. In this work, we have taken the term ischemic heart disease to be interchangeable with the commonly used term coronary heart disease [19].

### Search strategy

A systematic review was conducted to ascertain papers reporting on IHD associated with major depression that met predetermined inclusion and exclusion criteria (Table 1). Recommendations from the Preferred Reporting Items for Systematic Reviews and Meta-Analyses (PRISMA) Statement 2009 were taken into account throughout the study [23]. Data was sourced through a two-stage process. The first stage comprised a systematic search of the peer-reviewed literature. The next stage of the search involved identifying review articles, meta-analyses, editorials and resource books most pertinent to this disorder and examining the reference list of each to identify any further data sources. Data extracted from papers included study descriptors (for example, design, sample ascertainment, location, representativeness), sample descriptors (for example, age, gender, rural or urban), exposure and outcome parameters (for example, case definitions, diagnostic criteria, type of estimate, period of follow-up, estimate error) and confounding factors controlled for in the analysis. Further details of the search strategy and systematic review can be found in Additional file 1.

**Table 1:** Study inclusion and exclusion criteria

**Inclusion criteria**	**Exclusion criteria**
Meets the pre-determined case definitions of major depression and IHD	Cross-sectional study design
Longitudinal case–control or cohort study design	Clinical samples, for example, studies using hospital data
Sampling is from the general population	
Demographic characteristics of sample represent populations at risk	
Estimate of risk and associated uncertainty/error is reported, or sufficient data is reported to allow calculations of these	
Where multiple papers draw on the same sample, the most comprehensive or recent publication is preferred	
Subjects with clinical manifestations of IHD at baseline should be excluded or, at the very least, controlled or stratified for in the analysis	

### Meta-analysis

MetaXL software version 0.1 (http://www.epigear.com), a meta-analysis add-in for Microsoft Excel, was used to pool RR estimates from individual studies. A ‘quality effects model’ was chosen in addition to the random effects models to explicitly address heterogeneity between studies [24,25] (see Additional file 2 for quality checklist). The random effects model is usually chosen over a fixed effects model when there is significant heterogeneity across studies as it incorporates an estimate of between-study variation into the analysis [23]. Since the quality effects model weighs studies based on both the study quality and the sample size it is able to control for variability due to true differences in RR estimates and also differences due to study quality [17,18,26].

Sensitivity analyses were also conducted using MetaXL to test the effect of individual studies on the overall pooled estimate by removing one datapoint from the sample at a time. Meta-analyses were stratified by independent variables of interest, that is, sex and fatality (fatal vs non-fatal IHD event). Publication bias was investigated by means of funnel plots.

### Attributable burden estimation

The fundamental approach for the GBD 2010 comparative risk assessment is to estimate the proportion of deaths or disease burden caused by specific risk factors while holding other independent factors constant. Because most diseases are caused by multiple factors, and because some risk factors act through other, more proximal factors, population attributable fractions for multiple risk factors for the same disease can add to more than 100%. In other words, the burden attributable to different risks overlaps because of multicausality and because the effects of some risk factors are partly mediated through other, more proximal, risks [3,4].

Using counterfactual analysis, the effect of a risk factor can be quantified by comparing the burden associated to an outcome with the amount that would be expected in a hypothetical situation of ‘ideal’ risk factor exposure (in this case, a world without major depression). The end result is known as a population attributable fraction (PAF) [27-29]. This approach provides a consistent method for estimating the changes in population health as a function of decreasing or increasing the level of exposure to risk factors [29]. It should be noted that the terms population attributable risk (PAR) (expressed as a proportion) and PAF are synonymous and interchangeable [30]. For GBD purposes we will use PAF.

PAFs associated with major depression as a risk factor for IHD was calculated using the pooled RR estimates. For more information on the prevalence data, refer to the Mental Disorders Research Group’s reports on the methodology and results for compiling epidemiological data for mental disorders http://qcmhr.uq.edu.au/research/policy-and-epidemiology/peabod/burden-of-disease/. The PAF was calculated using the following equation:

$$\mathrm{PAF}=\frac{p\left(\mathrm{RR}-1\right)}{1+p\left(\mathrm{RR}-1\right)}$$

Where *P is* the prevalence of major depression and RR is the corresponding pooled relative risk estimate of IHD.

In the absence of evidence of differing risk between age, gender or morbidity versus mortality our pooled RR was applied across all groups. Prevalence data was estimated separately for each country, age, sex and year group, resulting in PAFs for each group [31]. In line with findings in the literature it was decided to apply the pooled RR to adults over 30 years of age only and as such no estimates are presented in this paper for persons less than 30 years of age.

Attributable burden is calculated by multiplying the PAF by the underlying burden. The burden due to IHD was estimated as part of the Global Burden of Disease Study 2010 [2]. Further details on the estimation of IHD burden are available elsewhere [21]. In order to reflect the uncertainty in our estimates, we calculated 1,000 draws each from the posterior distributions of the prevalence of MDD for each group, the relative risk, and the IHD burden for each group. We assumed independence between the uncertainty in these quantities and computed the attributable burden for each of the 1,000 sets. We report the mean and 2.5th and 97.5th centile values of these draws.

## Results

### Systematic search and meta-analysis

A total of 8 studies examining major depression as a risk factor for IHD met the inclusion criteria (Table 1) providing a total of 13 effect size estimates for analysis (see Additional files 3 and 4 for search flow diagram and summary of studies). The data represents observations from a collective sample size of over 35,000 subjects. One study was from The Netherlands [32], all other studies originated from the USA. Two studies were nationally representative [33,34]. Three studies were of older samples (over 50 years of age) [32,34,35]. The minimum length of follow-up was 4 years and extended to 37 years. Three studies employed a measurement of major depression that can be deemed truly diagnostic. The remaining studies all used the Center for Epidemiologic Studies Depression Scale (CES-D) and a symptom threshold to determine presence of major depression.

**Figure 1:**
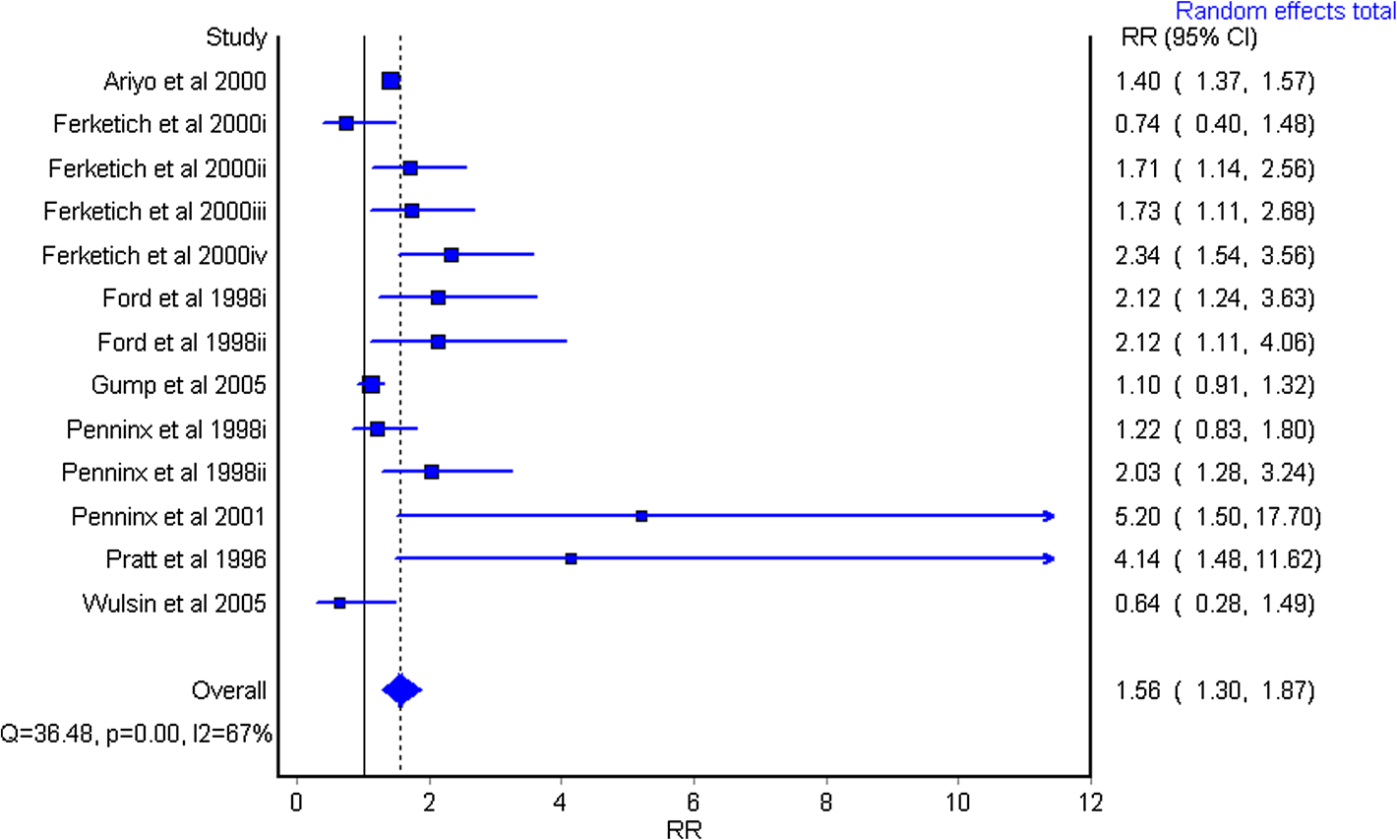
Pooled relative risk of incident ischemic heart disease (IHD) caused by major depression, random effects model.

Overall, major depression was associated with a significantly elevated risk of incident IHD. Using a random effects model, the pooled relative risk was 1.56 (95% CI 1.30 to 1.87) (Figure 1). There was little difference in results using the quality effects model (RR 1.54 (95% CI 1.27 to 1.87)), therefore we opted to continue analyses using the random effects model. Sensitivity analysis removing one datapoint from the sample at a time yielded RRs ranging from 1.49 (95% CI 1.24 to 1.89) to 1.66 (95% CI 1.34 to 2.04). All results remain statistically significant and show a comparably elevated risk for IHD. Three estimates where IHD case definitions included ICD 9 code 429 (ill-defined descriptions and complications of heart disease) or self-report were included in the analyses based upon reviewer consensus. Removal of these data points from the analysis demonstrated no impact on overall relative risk (1.56 (95% CI 1.28 to 1.89)).

A test for heterogeneity of the entire dataset demonstrates a relatively high degree of heterogeneity with an I^2^ statistic of 67.1% (*P* = 0.000). A possible publication bias was identified showing there may be some bias towards studies with positive results (see Additional file 5).

Stratification of risk estimates for a fatal IHD event versus a non-fatal outcome yielded non-significant differences (non-fatal IHD (RR 1.8, 95% CI 1.34 to 2.65), fatal events (RR 1.54, 95% CI = 0.85 to 2.80), and all events (RR 1.51, 95% CI 1.19 to 1.90). Further stratification of results, for example, by gender were severely limited by insufficient data; however, exploratory analyses indicated a large difference in effect size, dependent upon type of depression measurement tool, with clinical diagnosis inferring a greater relative risk for the development of IHD (RR 2.50, 95% CI 1.73 to 3.60 versus RR 1.40, 95% CI 1.17 to 1.68).

### Burden of disease

A summary of regional and global burden of disease estimates is presented in Table 2. Globally there were almost 4 million estimated IHD DALYs that can be attributed to major depression in 2010. The overwhelming majority of this burden is attributed to years of life lost (3.5 million years of life lost (YLL)) with a much smaller component of IHD morbidity (250,000 YLD). As a result, we now see a mortality component to major depression burden as a risk factor that was not apparent from estimates of the burden of major depression as a disease (YLL was estimated to be 0) [36].

**Table 2:** Attributable ischemic heart disease (IHD) burden estimates by region, 2010 (95% CI)

**Region**	**Disability-adjusted life years**	**Years of life lost**	**Years lived with a disability**
Asia Pacific, High Income	39,982 (19,701 to 66,462)	33,769 (16,218 to 56,464)	6,212 (2,413 to 12,936)
Asia, Central	112,925 (57,161 to 177,173)	108,331 (54,721 to 170,514)	4,593 (1,983 to 8,725)
Asia, East	386,610 (186,346 to 616,455)	347,277 (164,890 to 550,414)	39,332 (17,416 to 70,703)
Asia, South	863,351 (429,292 to 1,375,835)	829,281 (411,750 to 1,325,451)	34,069 (15,321 to 60,095)
Asia, Southeast	263,285 (135,382 to 421,193)	245,258 (125,893 to 395,411)	18,026 (7,955 to 32,258)
Australasia	9,622 (4,870 to 15,408)	8,541 (4,344 to 13,721)	1,081 (421 to 2,028)
Caribbean	29,484 (14,661 to 46,004)	27,773 (13,791 to 43,647)	1,710 (728 to 3,224)
Europe, Central	137,495 (69,706 to 214,634)	129,873 (65,902 to 202,094)	7,621 (3,384 to 14,119)
Europe, Eastern	664,145 (321,237 to 1,063,148)	639,605 (308,328 to 1,023,931)	24,540 (10,754 to 43,824)
Europe, Western	284,320 (144,755 to 435,735)	252,487 (127,659 to 387,169)	31,833 (13,996 to 56,870)
Latin America, Andean	12,761 (6,305 to 20,517)	11,495 (5,747 to 18,348)	1,266 (529 to 2,470)
Latin America, Central	84,115(43,308 to 132,587)	77,860 (39,465 to 122,414)	6,255 (2,652 to 12,031)
Latin America, Southern	31,373 (15,066 to 53,307)	28,547 (13,876 to 47,362)	2,825 (1,140 to 5,412)
Latin America, Tropical	127,581 (65,215 to 208,633)	115,548 (58,951 to 191,580)	12,032 (5,224 to 21,858)
North Africa/Middle East	363,828 (183,937 to 571,914)	342,820 (171,720 to 533,404)	21,008 (9,630 to 37,854)
North America, High Income	244,716 (125,676 to 394,058)	223,728 (114,409 to 358,292)	20,987 (9,588 to 38,059)
Oceania	3,628 (1,751 to 6,339)	3,355 (1,623 to 5,891)	272 (107 to 507)
SubSaharan Africa, Central	28,336 (13,710 to 47,204)	26,682 (12,849 to 44,673)	1,653 (667 to 3,421)
SubSaharan Africa, East	62,438 (31,898 to 99,496)	55,098 (28,007 to 88,043)	7,340 (3,205 to 13,455)
SubSaharan Africa, Southern	18,385 (9,071 to 30,166)	16,567 (8,175 to 27,355)	1,817 (771 to 3,341)
SubSaharan Africa, West	54,615 (27,511 to 86,304)	48,864 (24,879 to 76,381)	5,750 (2,483 to 11,008)
Global	3,823,004 (1,942,771 to 5,778, 350)	3,572,770 (1,791,433 to 5,411,987)	250,233 (114,845 to 444,063)

Absolute IHD DALYs attributable to major depression in 2010 ranged from approximately 4,000 DALYs in Oceania to 900,000 in South Asia. The lowest regions across all three timepoints were Oceania, Australasia and Latin America, Andean (see Additional file 6). The highest were Eastern Europe and South Asia, this being driven by large IHD burden and large population size, respectively. Importantly for all regions, uncertainty ranges for each timepoint overlap.

**Figure 2:**
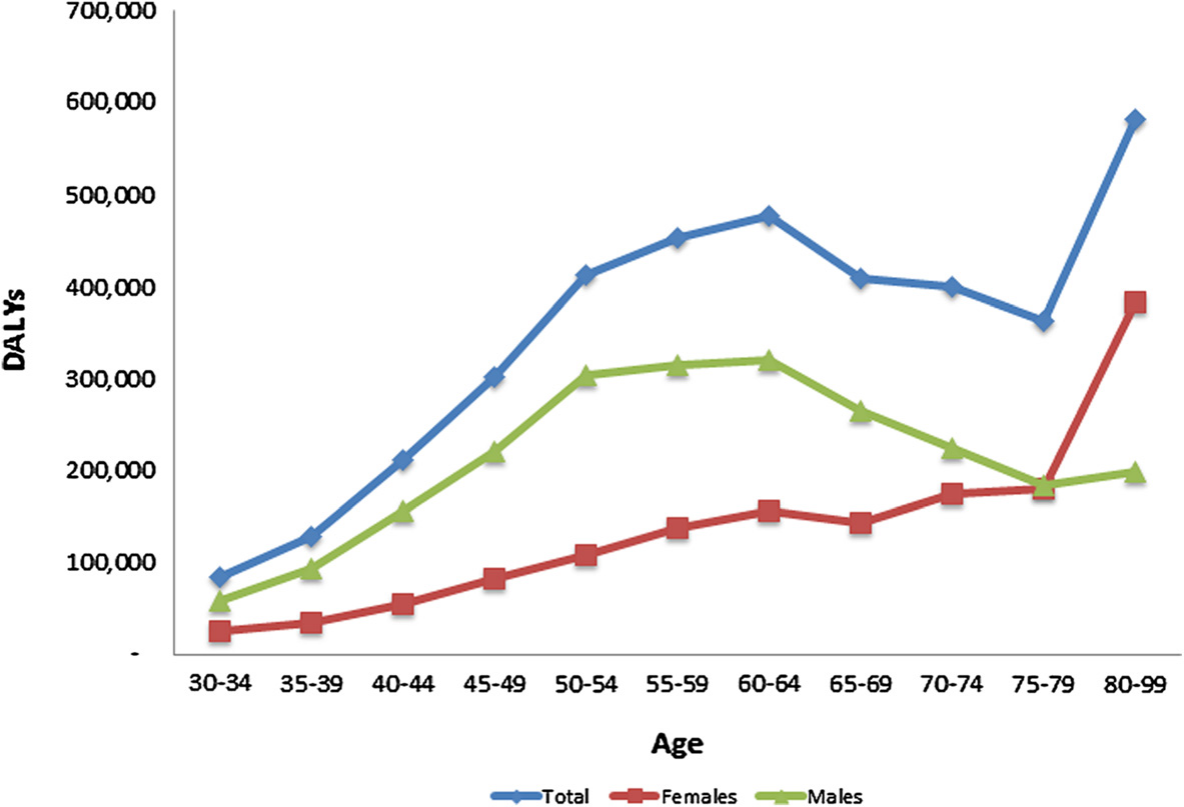
Global ischemic heart disease (IHD) disability-adjusted life years (DALYs) attributable to major depression by age and sex, 2010.

The overall age pattern shows a steady increase in attributable burden that peaks at around 60 years of age (Figure 2). The sharp rise in attributable DALYS in the older ages is reflective of the significant rise in IHD burden in this group [37]. As expected, males demonstrate higher burden of IHD attributable to major depression than females; however, this is only maintained until around age 80. Note the apparent sudden increase in DALYs after age 80 in females is an artifact of an 80+ age group (as opposed to a 5-year age group). The drop in burden seen at around 60 years of age for males is explained by a moderately high (and peak) IHD death rate in middle age coupled with large population at risk at those ages, despite a higher IHD death rate at older ages. Female IHD risk accelerates around age 65 years and explains the late rise in DALYs. It is important to note that uncertainty bounds around male and female estimates are large and overlapping.

**Figure 3:**
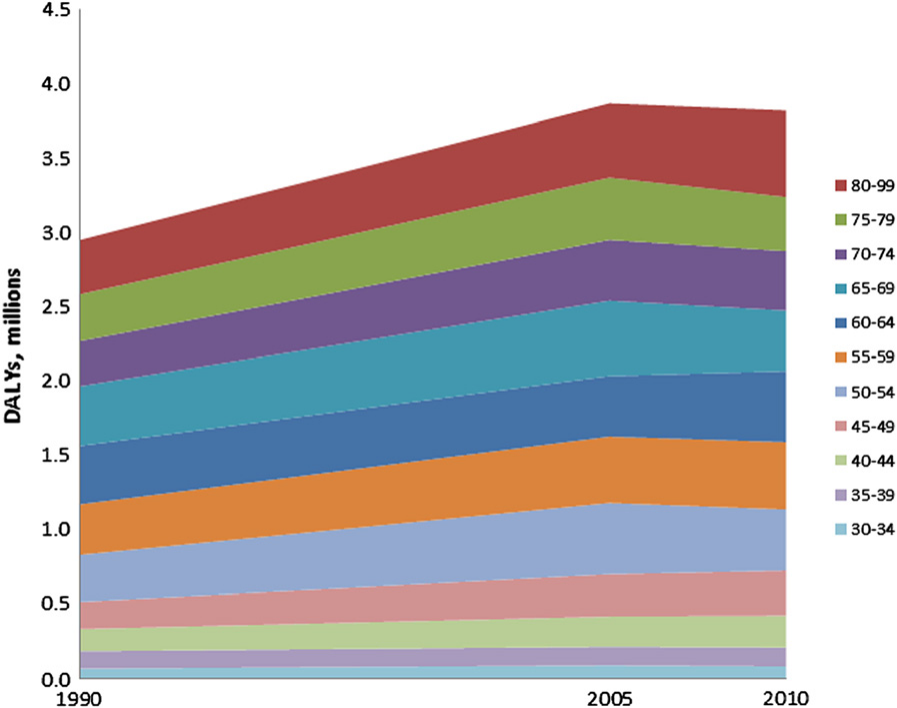
Change in ischemic heart disease (IHD) disability-adjusted life years (DALYs) attributable to major depression over time, by age.

The IHD burden attributable to major depression has increased over time from 3.0 million DALYs in 1990 to 3.8 million DALYs in 2010 (Figure 3). However, the majority of this increase occurred in the 1990 to 2005 period with an apparent stabilization of burden between 2005 and 2010. This overall increase is reflective of an increase in IHD DALYs largely driven by population growth and aging [37].

Mirroring observations made in IHD deaths, the over 80s contribute most to the burden increase [37]. Nearly 50% of DALYs are in the over 65s and approximately 80% in over 50s.

**Figure 4:**
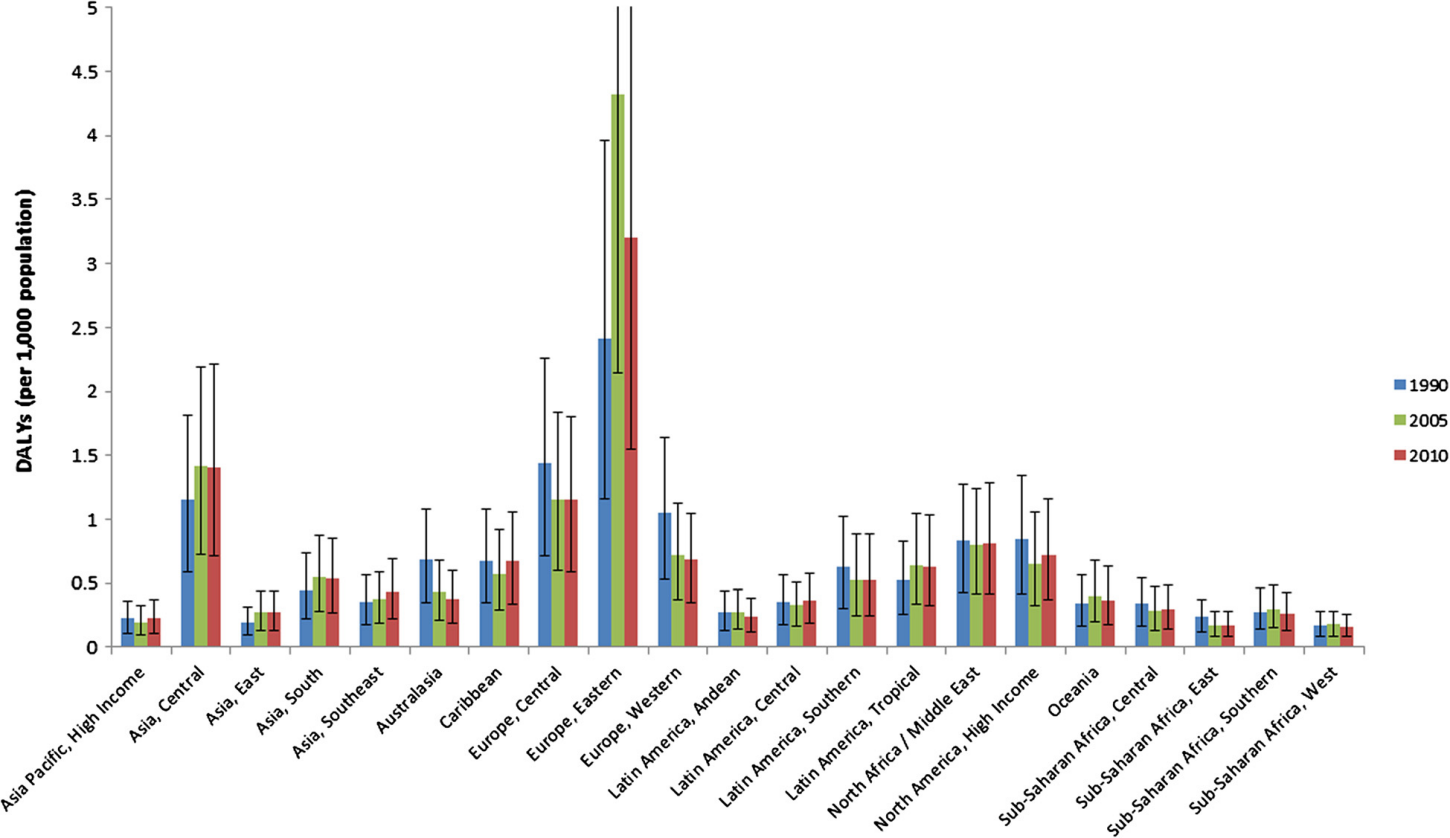
Ischemic heart disease (IHD) disability-adjusted life year (DALY) rates (per 1,000 population) attributable to major depression by world region for 1990, 2005 and 2010 (age and sex standardized).

Presenting IHD DALYs attributable to major depression as an age-standardized and sex-standardized rate facilitates comparisons between regions (Figure 4). There appears a disproportionate burden in Eastern Europe, Central Europe and Central Asia with sub-Saharan Africa, East and West falling to the lower rankings (see Additional file 7).

Time trends are not consistent across regions with some regions experiencing an increase in DALY rates across the timepoints and others experiencing a decline (Figure 4). The exceedingly high rates seen in Eastern Europe and Central Asia (former Soviet Union states) are partnered with an increase over time. Increasing trends over time are also observed in the remaining Asian regions (South, East and Southeast), Oceania and parts of Latin America. Encouragingly, all other regions have demonstrated a reduction in attributable burden since 1990.

**Figure 5:**
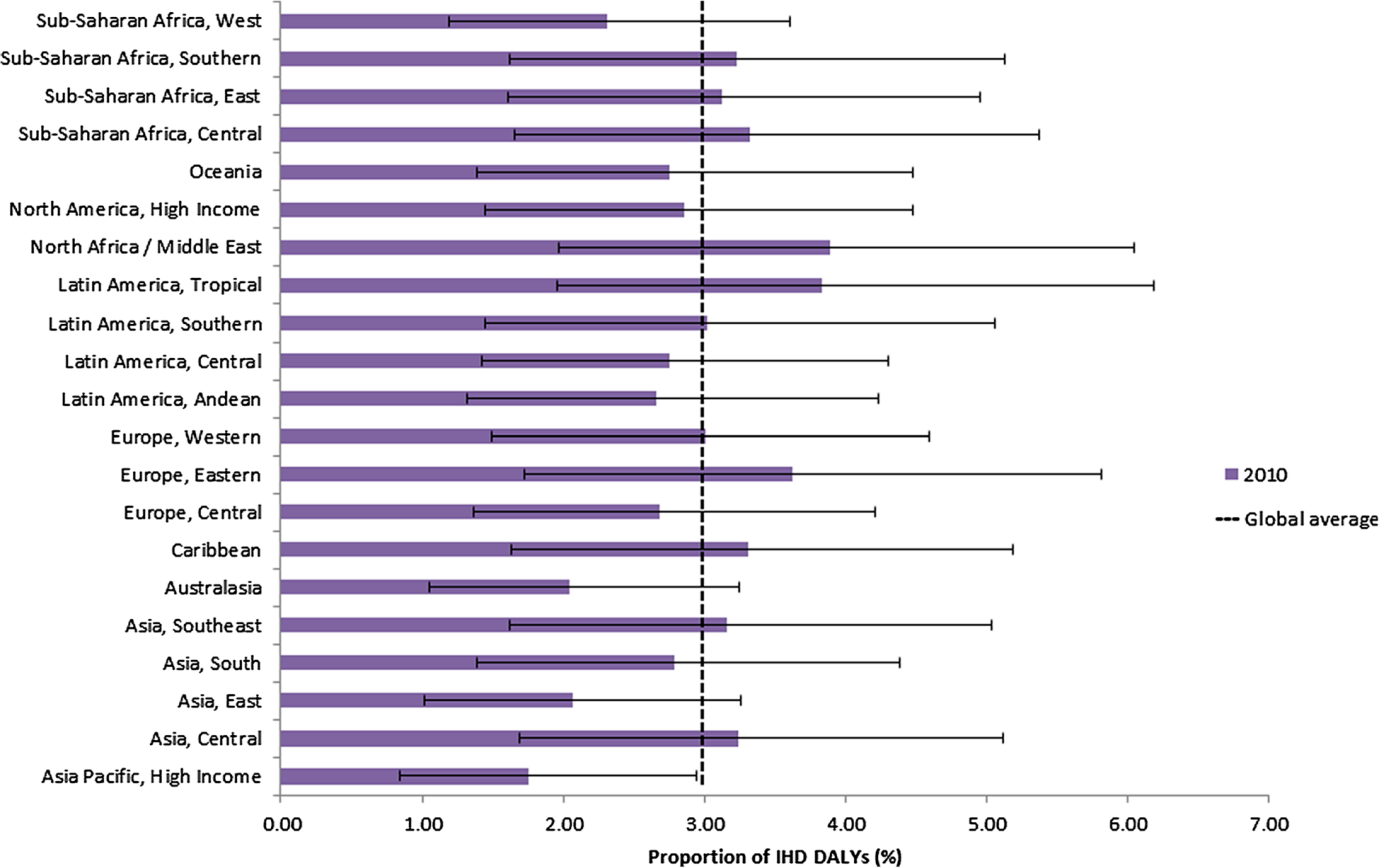
Ischemic heart disease (IHD) disability-adjusted life years (DALYs) attributable to major depression as a proportion of overall IHD DALYs by Global Burden of Disease (GBD) study region, 2010.

As a proportion of overall IHD burden, 2.95% (95% CI 1.48% to 4.46%) of IHD DALYs were estimated to be attributable to MDD in 2010. Eastern Europe and North Africa/Middle East demonstrate the highest proportion with Asia Pacific, high income representing the lowest (Figure 5). These percentage estimates are reflective of a function of both regional IHD prevalence and mortality and major depression prevalence patterns where a higher prevalence of MDD in the population will result in a higher proportion of IHD cases being attributable to major depression. For this reason we see the higher MDD prevalence regions of North Africa/Middle East, Eastern Europe and large areas of sub-Saharan Africa and Latin America also experiencing the highest proportion of IHD cases being attributable to major depression [31]. Conversely, Asia-Pacific, high income, Australasia, and Asia East were estimated to have the lowest prevalence of major depression. The attributable burden age patterns are also strongly influenced by trends in major depression prevalence (not shown) [31].

**Figure 6:**
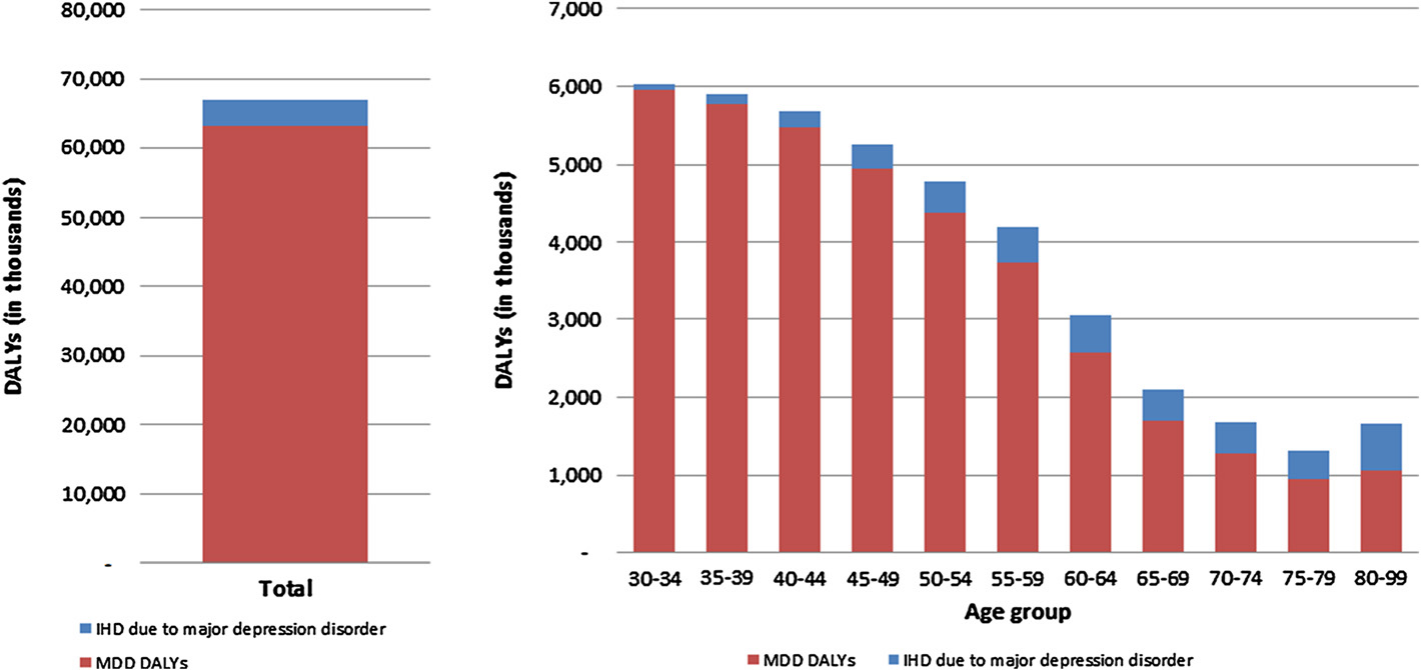
Cumulative burden of disease of major depression, 2010.

The IHD burden attributable to major depression is largely due to IHD mortality. This indirectly adds a mortality component to the burden of major depression seemingly missing from the burden of major depression [36]. Reassigning attributable DALYs to the direct burden of major depression creates a cumulative burden of almost 70 million DALYs (Figure 6). As a proportion of global DALYS, major depression can be revised upwards to 2.7% from 2.5%.

## Discussion

We found a significant increase in risk of developing IHD in people suffering from major depression (pooled RR 1.56 (95% CI 1.30 to 1.87)). Almost 4 million, or 3% of IHD DALYs were attributable to major depression in 2010; the majority of which resulted from premature death. These findings highlight a previously underestimated mortality component of the burden of major depression.

We used findings from a systematic review and meta-analysis of observational studies using predetermined inclusion criteria which attempt to delineate the independent relationship of depression leading to incident IHD from the complex and bidirectional, biological and behavioral network as described by Stapelberg *et al*. [38,39]. Key differences and strengths over previous reviews are the inclusion of only longitudinal studies which exclude, or control for baseline IHD, as well as measures of depression which closely approximate clinical major depression. Only studies with samples that could be considered acceptably representative of the general population were accepted for inclusion. These criteria are essential in order to most accurately assess any mental disorder as an independent risk factor for IHD in the general population.

### Findings

All studies that were identified and met our criteria were prospective cohort studies that were randomized or included the entire target population. These provided evidence of a temporal relationship for major depression preceding IHD, and this is relevant in terms of the validity of our findings as cross-sectional studies of the relationship between depression and IHD have been shown to overestimate the magnitude of the association [12].

The increased risk (RR 1.56, 95% CI 1.30 to 1.87) found in this review support previous findings. Four earlier meta-analyses reported pooled relative risk for depression and incident IHD between 1.57 and 2.69 [6,12,40,41], and major studies such as INTERHEART [42], which found an OR of 1.44 (95% CI 1.27 to 1.65) for incident MI.

Statistical power was insufficient for robust subgroup analyses but a notable finding from exploratory analyses was a large difference in effect size, dependent upon type of depression measurement tool, with clinical diagnosis inferring a greater relative risk for the development of IHD. This could be a reasonable expectation given that screening tools are likely to capture subthreshold cases of depression, which may pose a lower risk of developing incident IHD. Indeed, a dose–response relationship and a higher risk of IHD in depression meeting diagnostic thresholds versus symptoms have been shown. The use of a relative risk attained by pooling diagnostic instrument obtained estimates would have led to PAFs 2.7 times higher than those calculated in this paper resulting in a large underestimation of attributable burden.

The disproportionate IHD burden attributable to major depression that is carried by Eastern Europe, Central Europe and Central Asia infers the need for priority attention, particularly given the large IHD burden seen in these regions. The higher proportions of IHD DALYs seen in North Africa/Middle East and areas of sub-Saharan Africa and Latin America also warrant attention where reductions in the prevalence of major depression could reduce the overall burden of IHD attributable to major depression.

Time trends are not consistent across regions with some regions experiencing an increase in burden across the timepoints and others experiencing a decline. It is important to note that increases in burden are largely driven by population growth and ageing, not changes in actual prevalence rates.

Importantly, these findings add to the incomplete picture of direct burden for major depression, which did not include YLL [36]. Our study shows that indirect pathways of major depression are not only responsible for significant morbidity but also contribute to premature mortality via other health outcomes.

### Limitations

Estimating the proportion of IHD attributable to major depression implies a causal relationship between depression and IHD. This assumption remains to be definitively established. Despite the body of evidence reviewed here demonstrating an association between depression and IHD, and observational evidence fulfilling some of the criteria for causality, the behavioral and biological drivers of the association remain poorly defined, largely due to the complex nature of depression and IHD, as well as the complexity of the relationship between the two diseases [39]. While a causal relationship between depression and coronary heart disease is proposed, there are several related risk factors, for example, smoking, diet, alcohol consumption, physical activity and obesity, which likely explain at least part of the association, supported by the fact that a bidirectional relationship exists between them [16].

A further potential confounder might also be comorbid anxiety [39,43], which itself has a significant relationship with IHD [43]. Further work is needed to quantify the relationship between anxiety disorders and IHD. Generalized anxiety disorder (GAD) is known to co-occur in 30% to 40% of people with MDD between the ages of 18 and 65 years [29] and in older people aged 55 to 85 years, 47.5% of those with a MDD also meet the criteria for at least one anxiety disorder [44]. A meta-analysis of 20 studies on the association between anxiety alone and the risk of incident IHD reported a hazard ratio of 1.26 (95% CI: 1.15 to 1.38; *P* <0.0001) and cardiac death hazard ratio of 1.48 (95% CI: 1.14 to 1.92; *P* = 0.003), independent of demographic variables, biological risk factors, and health behaviors [45]. Anxiety and MDD together were also shown predict the risk of major adverse cardiac events risk in patients with IHD [46]. GAD has further been shown to be a significant confounder in specific methods used to investigate the relationship between MDD and IHD, such as heart rate variability (HRV) [39].

Importantly, one piece of evidence, the hypothesis that successful depression treatment lowers IHD risk, has not been proved despite enormous efforts [47]. The Enhancing Recovery in CHD Patients (ENRICHD) randomized controlled trial in acute coronary syndrome patients found no greater IHD-risk reduction in the treated depression arm compared with the control depression group [48]; other trials were not powered to detect change in IHD risk [49]. Ongoing trials, such as the Comparison of Depression Interventions after Acute Coronary Syndrome (CODIACS) trial will hopefully test the hypothesis that depression treatment lowers IHD risk more definitively [50].

In this review and CRA assessment, one study was from The Netherlands and all other studies originated from the USA. Only two studies were shown to be nationally representative. This lack of representativeness makes it difficult to generalize results or determine risk estimates, population attributable fractions, and attributable risk for populations in other age groups, countries or regions. Nevertheless, we elected to apply the pooled relative risk from these two regions to estimate the disease burden of IHD attributable to major depression worldwide. An underlying assumption of using a consistent measure of risk is that the calculated relative risk is invariant across countries. Such invariance could be considered plausible given evidence suggesting that mechanisms which underlie the relationship between MDD and IHD are primarily biological [6], as are the changes in behavior associated with MDD, previously thought to be psychologically driven [51-58], however without data this cannot be confirmed. The alternative to applying a universal relative risk across all regions is to exclude 19 of the world’s 21 GBD regions from estimations of attributable burden, however the decision to estimate attributable burden for regions where there was no data has been a standard position taken by the Global Burden of Disease Study 2010 [59,60]. While contentious, the view is taken that to not provide estimates for regions with missing data is to infer that burden does not exist. The implications of this are considered unacceptable and estimates have been produced while simultaneously calling for action in the research community to fill data gaps [61].

The lack of targeted risk assessment research in this area is highlighted by the fact that most data found for this review originates from a secondary analysis of large population cohort studies with different primary aims potentially leading to sources of bias. There was significant heterogeneity between studies (I^2^ = 67.1%) largely due to differences in study design, which makes the undertaking of study comparison and pooling of data difficult and limits conclusions that can be drawn. The significant heterogeneity in study design that exists in this field of study has been identified in the literature [62]. Additionally, there is also the potential for overestimation of effect size due to the apparent publication bias towards positive studies.

While any bias due to errors in measurement of major depression could have been reduced by only accepting clinical diagnosis or diagnostic interviews, the few studies that met this criterion would have made the current review unfeasible. Very few of the studies identified in our systematic review use well-validated instruments that reliably provide a diagnosis of major depressive disorder. An improvement in the measurement of depression is an area of opportunity for future research.

GBD 2010 did not include ‘silent’ IHD in its case definition because for the purposes of the study only symptomatic (that is, disabling) diseases were measured. No studies identified by our review included ‘silent’ IHD. Because some cases of ‘silent’ IHD were possibly classified in the ‘no IHD’ group in these studies, the implication is that these studies underestimated the association between IHD and depression, making our estimates overly conservative. These key limitations have been discussed elsewhere [46].

Given the gender differences in both MDD and IHD [31,37] and confounding effect of age [63,64], we aimed to stratify risk by these characteristics. However, dearth of data did not allow this, necessitating the application of a summary RR to both males and females across all age groups (older than 30 years of age). Although it could be reasonable to expect a higher risk for non-fatal events over fatal events, the same data restrictions meant the one RR was applied uniformly to the development of both YLD and YLL.

### Implications

Interactions between mental disorders and a range of other health outcomes have been widely recognized in the literature yet mental health is largely missing from relevant public health policy framework, research priorities and targets for interventions [10]. Given the accumulating evidence for the role that mental disorders appear to play in non-communicable disease, the low treatment rates seen globally warrant review [65].

The *European Psychiatric Association* aims to increase the awareness among psychiatrists and primary care physicians caring for patients with severe mental illness about the need to screen and treat increased cardiovascular risk factors and diabetes [66]. However, more conclusive evidence is needed about the effect of treatment of mental disorders such as depression in helping decrease the risk of non-communicable disorders such as IHD. Nonetheless, the complex and bidirectional relationships between comorbid mental disorders and cardiovascular disease require the care for persons with these conditions to be coordinated and collaborative [67].

## Conclusions

This paper comprises the most robust systematic review of its kind to date and is one of only two comparative risk assessments assessing major depression as an independent risk factor for another health outcome [68]. It highlights the significant association between major depression and incident IHD, as well as quantifying the impact of depressive illness as an independent risk factor for IHD.

To establish the patterns and nature of the depression and IHD relationship, the body of evidence needs strengthening. Deconstructing the causal network linking depression and IHD is crucial [38,69]. A true understanding of behavioral and biological pathways, the interrelationship between different risk factors, and mental disorder comorbidity issues, is needed [19].

Furthermore, there is a need for further well-designed and targeted research examining mental disorders as independent risk factors for IHD, particularly in regions outside of North America, and for the effect of mental health interventions on incident IHD. Differential risk levels by gender and age need to be explored in greater detail.

## Abbreviations

AB: Attributable burden; CES-D: Center for Epidemiologic Studies Depression Scale; CRA: Comparative risk assessment; DALY: Disability-adjusted life year; DSM: *Diagnostic and Statistical Manual of Mental Disorders* (American Psychiatric Association); GAD: Generalized anxiety disorder; GBD 2010: Global Burden of Disease Study 2010; HPA: Hypothalamic pituitary adrenal; HRV: Heart rate variability; ICD: *International Classification of Diseases* (World Health Organization); IHD: Ischemic heart disease; MDD: Major depressive disorder; NOS: Not otherwise specified; PAF: Population attributable fraction; PAR: Population attributable risk; WHO: World Health Organization; YLD: Years lived with a disability; YLL: Years of life lost.

## Competing interests

The authors declare that they have no competing interests.

## Authors’ contributions

FC: made a substantial contribution to conception and design of paper, acquisition and interpretation of data, played a principal role in drafting the article and revising it critically for important intellectual content and performed final critical edit, gave approval of the version to be published. AM, RN, CS, AB, TV and HW: made a substantial contribution to conception and design of paper, acquisition and interpretation of data, played an important role in drafting the article and revising it critically for important intellectual content, performed final critical edit and gave approval of the version to be published. GF: conducted statistical analyses and burden of disease calculations, performed final critical edit and gave approval of the version to be published. All authors read and approved the final manuscript.

## Pre-publication history

The pre-publication history for this paper can be accessed here:

http://www.biomedcentral.com/1741-7015/11/250/prepub

## Supplementary Material

Additional file 1 Description of search strategy and systematic review methodology. Click here for file

Additional file 2 Quality scoring checklist for meta-analysis using the quality effects model. Click here for file

Additional file 3 Search flow diagram. Click here for file

Additional file 4 Table of studies meeting selection criteria. Click here for file

Additional file 5 Funnel plot of included studies. Click here for file

Additional file 6 Absolute ischemic heart disease (IHD) disability-adjusted life years (DALYs) (in 1,000 s) attributable to major depression by world region for 1990, 2005 and 2010. Click here for file

Additional file 7 **World map showing regional attributable burden as: (A) disability-adjusted life years (DALYs) per 1,000 population (95% CI) and (B)percentage of overall ischemic heart disease (IHD) DALYs, for 2010.** Click here for file
