# Supplementary material for: The contribution of major depression to the global burden of ischemic heart disease: a comparative risk assessment
Source: BMC Med. 2013 Nov 26;11:250. doi: 10.1186/1741-7015-11-250 (PMC4222499; doi:10.1186/1741-7015-11-250)
Supplement: Additional file 1 — Description of search strategy and systematic review methodology. [file 1741-7015-11-250-S1.docx]

**Search strategy and systematic review methodology**

Data was sourced through a two-stage process. The first stage comprised a systematic search of the peer-reviewed literature. The search string was developed in collaboration with a research librarian and used to interrogate electronic databases (EMBASE and Medline). Terms used were “mental disorders’ AND (“ischaemic heart disease” OR “stroke”). An explosion search was conducted with all sub-terms and derivatives included in the search. No restrictions were set for year of publication or language.

The next stage of the search involved identifying review articles, meta-analyses, editorials and resource books most pertinent to this disorder and examining the reference list of each to identify any further data sources. Article titles were scanned for relevance and abstracts of pertinent articles were read in full to further cull the list according to the inclusion and exclusion criteria (Box).

Identified articles were imported into an Endnote database and duplicates deleted. Where multiple articles reported the same data, the most recent or informative article was used with duplicated reports excluded. Article titles were scanned for relevance and abstracts were read to filter for those containing epidemiologic data. The full-text article of any study that appeared to meet the inclusion criteria was retrieved and examined by two independent reviewers. Individual-level case-control and prospective cohort studies were considered the gold standard in assessing the directional relationship between exposure to major depression and incident IHD. Case series, or retrospective studies were not included.

*Data extraction and quality assessment*

Data extracted from papers included study descriptors (e.g. design, sample ascertainment, location, representativeness), sample descriptors (e.g. age, gender, rural or urban), exposure and outcome parameters (e.g. case-definitions, diagnostic criteria, type of estimate, period of follow-up, estimate error) and confounding factors controlled for in the analysis. For major depression, risk estimates were based on whether individuals were classified as a case or not a case as defined above. Where a dichotomous measure of major depression ‘caseness’ or a symptom scale cut-point was not provided for identifying the presence of major depressive symptoms, the relative risk of the highest score (most severe) group over the lowest was used. Relative risk (RR), hazard ratio (HR) and odds ratio (OR) estimates were deemed to approximate each other and appropriate to combine for data analysis in this context [[60](#_ENREF_60)]. Each paper was examined to ensure no study contributed estimates based on overlapping samples; for example, where a study reported male, female and overall risk, only the male and female estimates were included. Similarly, where a study reported fatal, non-fatal and overall risk only the fatal and non-fatal estimates were included. A random sample of articles was double-checked for accuracy and consistency of data extraction and entry. If required, authors were also contacted for clarification of specific issues.

A quality index was used to quantify the methodological quality of each study. The quality index was made up of variables describing key areas of the study methodology such as the ascertainment site; sample and the methodology used for analysis. Studies were first scored based on how well they addressed each quality variable. Scores for each variable were then summed into a total quality index score for each study. Each quality score was expressed as a proportion (refer Appendix 5).
