# Supplementary material for: The contribution of major depression to the global burden of ischemic heart disease: a comparative risk assessment
Source: BMC Med. 2013 Nov 26;11:250. doi: 10.1186/1741-7015-11-250 (PMC4222499; doi:10.1186/1741-7015-11-250)
Supplement: Additional file 2 — Quality scoring checklist for meta-analysis using the quality effects model. [file 1741-7015-11-250-S2.pdf]

Major depression as a risk factor for IHD – quality scoring checklist for meta-analysis using the quality effects model

1. Was a method of randomization performed?

0 _ No or not reported

1 _ In Part

2 _ Yes

2. Was there a clinical diagnosis of major depression made?

0 _ No or not reported

1 _ In Part

2 _ Yes

3. Was there a clear case ascertainment of IHD reported?

0 _ No description

0.5 _ Self report or description in Part

1 _ Yes

4. Did the measured outcome include all categories of IHD (as defined in our study methodology)?

0.5 _ Measured only sub-categories then used as proxy for IHD e.g. MI

1 _ Yes

5. Were the important prognostic indicators of the group/cohorts comparable at baseline? e.g. medication use, age

0 _ No or not reported

0.5 _ In Part

1 _ Yes

6. How representative of the general population was the cohort from which the sample was drawn?

0 _ No or no description of the derivation of the cohort

0.5 _ In Part, or selected group of users (e.g., nurses, volunteers)

1 _ Yes

7. Did the sample include all ages in which IHD would typically present (i.e. >30)?

0 _ No, narrow age bracket only

1 _ Yes

8. Was the IHD already present at the start of study?

0 _ Yes

1 _ No

9. Were protocol deviations, losses to follow-up, and drop-out rates acceptable (<20%)?

0 _ No or not reported

0.5 _ In Part

1 _ Yes

10. Was length of follow-up comparable and adequate for outcomes to occur?

0 _ No or not reported

0.5 _ In Part

1 _ Yes

11. Was the analysis clear and appropriate?

0 _ No or not reported

0.5 _ In Part

1 _ Yes

Total score: Qi _ Sum of above
