# Supplementary figures and images for: The contribution of major depression to the global burden of ischemic heart disease: a comparative risk assessment
Source: BMC Med. 2013 Nov 26;11:250. doi: 10.1186/1741-7015-11-250 (PMC4222499; doi:10.1186/1741-7015-11-250)

## Search flow diagram

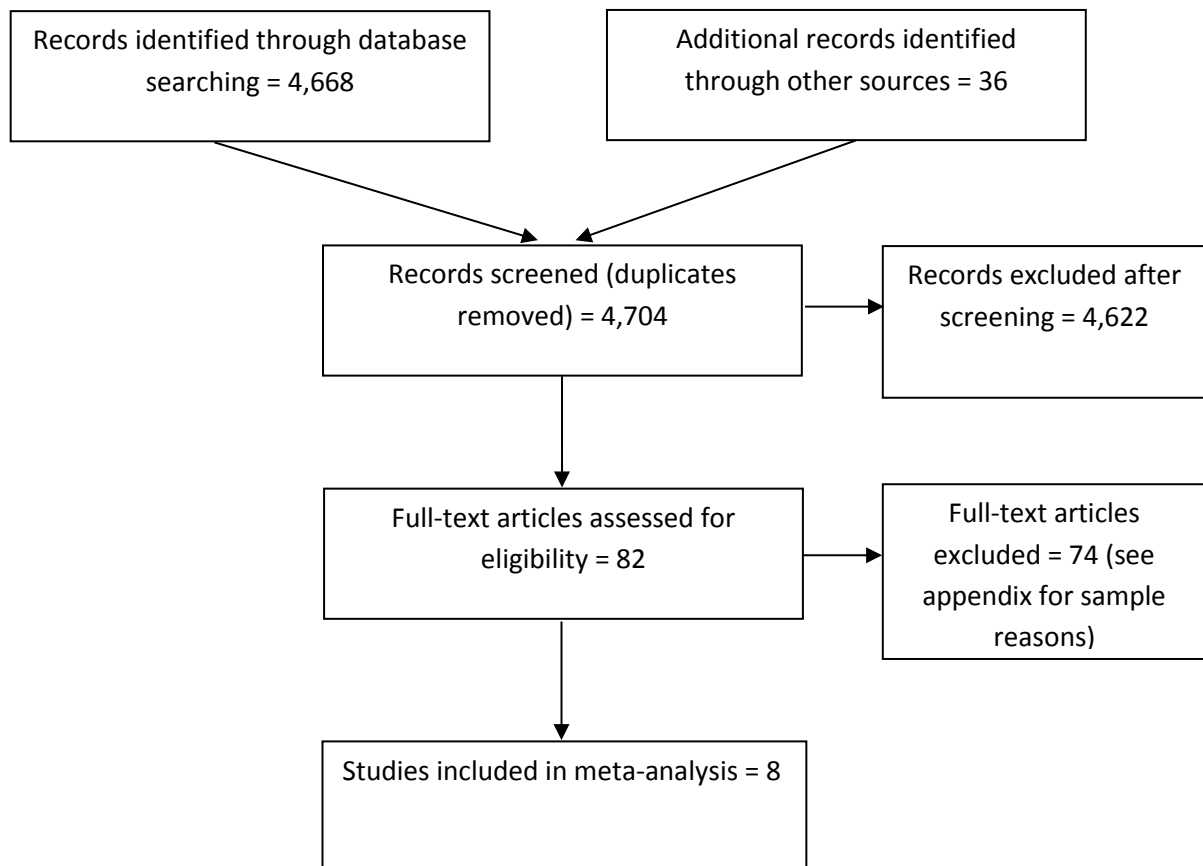

Supplement: Additional file 3 — Search flow diagram. [file 1741-7015-11-250-S3.pdf]

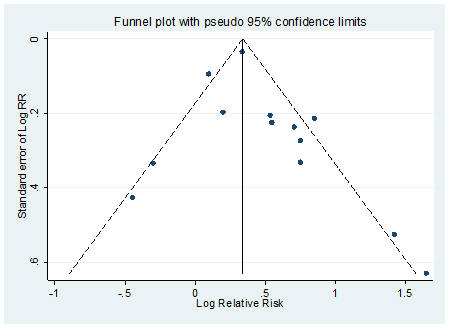

Supplement: Additional file 5 — Funnel plot of included studies. [file 1741-7015-11-250-S5.png]

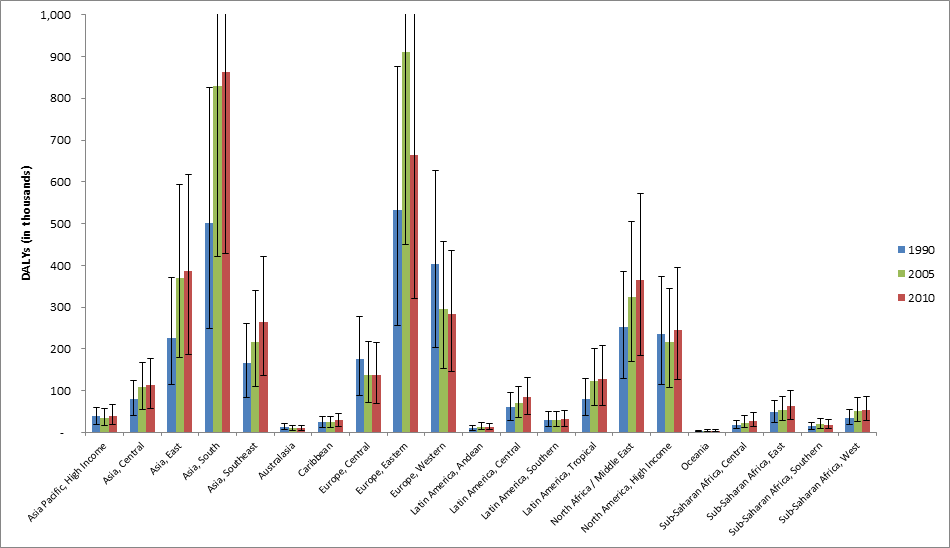

Supplement: Additional file 6 — Absolute ischemic heart disease (IHD) disability-adjusted life years (DALYs) (in 1,000 s) attributable to major depression by world region for 1990, 2005 and 2010. [file 1741-7015-11-250-S6.png]

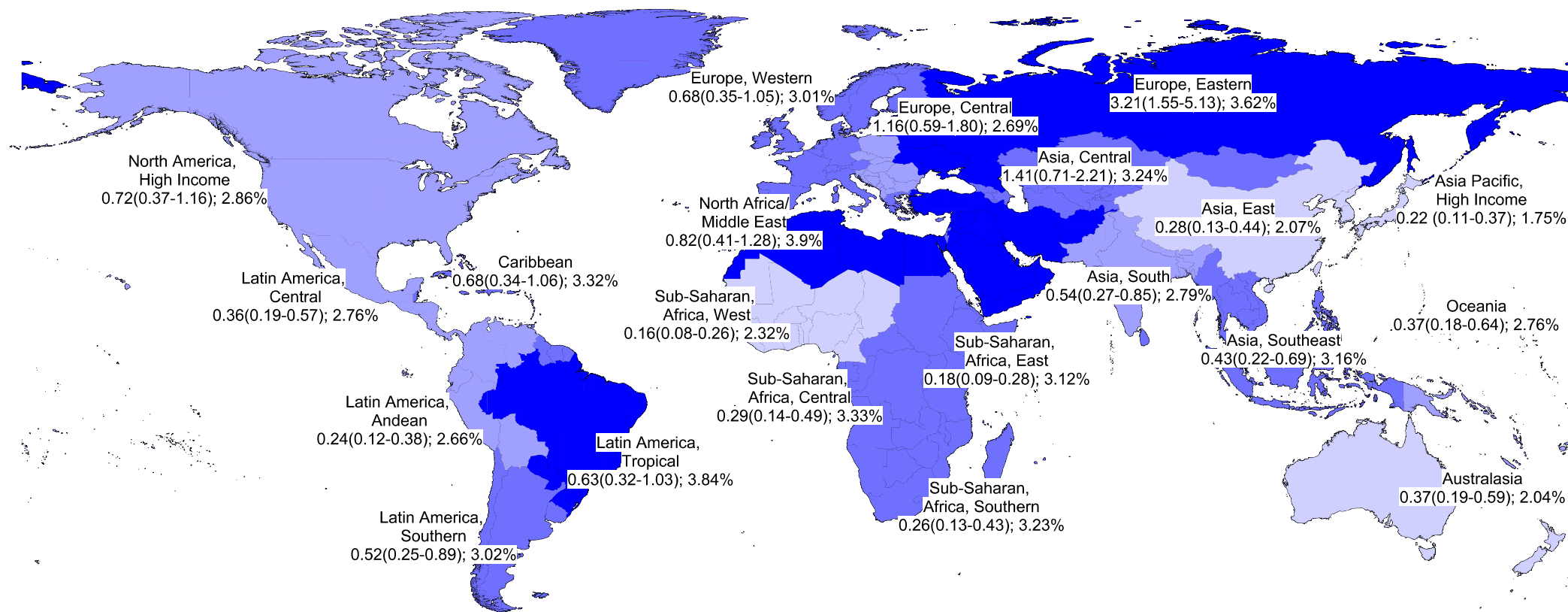

1.75-2.50%
  2.51-3.00%
  3.01-3.50%
  3.51-3.90%

Supplement: Additional file 7 — World map showing regional attributable burden as: (A) disability-adjusted life years (DALYs) per 1,000 population (95% CI) and (B)percentage of overall ischemic heart disease (IHD) DALYs, for 2010. [file 1741-7015-11-250-S7.pdf]
