# Supplementary material for: The contribution of major depression to the global burden of ischemic heart disease: a comparative risk assessment
Source: BMC Med. 2013 Nov 26;11:250. doi: 10.1186/1741-7015-11-250 (PMC4222499; doi:10.1186/1741-7015-11-250)
Supplement: Additional file 4 — Table of studies meeting selection criteria. [file 1741-7015-11-250-S4.docx]

**Appendix 3: Studies meeting selection criteria**

| Reference | Country | Epoch range | Exposure measurement method | Study population | Age range | N | Estimates |
| --- | --- | --- | --- | --- | --- | --- | --- |
| ([Ariyo AA and al. 2000](#_ENREF_1)) | US | 1989-1996 | CES-D | Cardiovascular Health Study. General population. | 65+ | 4493 | HR; incident IHD = 1.4 (95%CI = N/A); |
| ([Ferketich AK 2000](#_ENREF_2)) | US | 1982-1992 | CES-D | General population. | 30+ | 5007 women, 2886 men | Women: RR; non-fatal IHD events = 1.73 (95%CI = 1.11–2.68); fatal IHD events = 0.74 (95%CI = 0.40–1.48)  Men: RR; non-fatal IHD events = 1.71 (95%CI = 1.14–2.56); fatal IHD events = 2.34 (95%CI = 1.54–3.56) |
| ([Wulsin, Evans J.C. et al. 2005](#_ENREF_8)) | US | 1983-1989 | CES-D | Framingham study. Community-based. | 30-91 | 3634 | RR for incident IHD; dichotomous score (≥16) = 0.64 (95%CI = 0.28–1.49); continuous score = 0.99 (95%CI = 0.96–1.03) |
| ([Pratt LA 1996](#_ENREF_7)) | US | 1981-1994 | DIS | Baltimore ECA Follow-up Study. General population | 18+ | 1551 | OR for incident MI = 4.54 (95%CI = 1.65–12.44) |
| ([Penninx, Beekman et al. 2001](#_ENREF_5)) | Netherlands | 1992-1997 | CES-D and DIS | Longitudinal Aging Study Amsterdam (LASA). Community-based. | 55-85 | 2847 | RR for IHD mortality = 5.2 (95%CI = 1.5–17.7) |
| ([Penninx BWJH and Visser M 1998](#_ENREF_6)) | US | 1988-1992 | CES-D | 3 communities of the Established Populations for Epidemiologic Studies of the Elderly (EPESE). | 70+ | 3701 | Incident IHD: Men: RR = 2.03 (95%CI = 1.28–3.24). Women: RR = 1.22 (95%CI = 0.83–1.80). Total: RR = 1.47 (95%CI = 1.10–1.98) |
| ([Gump B.B., Matthews K.A. et al. 2005](#_ENREF_4)) | US | 1973-1999 | CES-D | Multiple Risk Factor Intervention Trial (MRFIT). Men free of IHD but with above average risk of IHD. | 35-57 | 11263 | HR of 5^th^ quintile; IHD mortality = 1.10 (95%CI = 0.91–1.32); cerebrovascular mortality = 2.03 (95%CI = 1.20–3.44) |
| ([Ford DE 1998](#_ENREF_3)) | US | 1948-1964 | Self-report confirmed by physician review panel | John Hopkins Precursors Study. All male students enrolled in John Hopkins Medical School | N/A | 1190 | RR of incident IHD = 2.12. (95%CI = 1.24 – 3.63); RR of incident cerebrovascular accident = 0.93 (95%CI = 0.33 – 2.65) |

Ariyo AA, H. M., Tangen CM, Rutledge JC, Cushman M, Dobs A and e. al. (2000). "Depressive symptoms and risks of coronary heart disease and mortality in elderly Americans." Circulation **102**: 1773-1779.

Ferketich AK, S. J., Frid DJ, Moeschberger ML. (2000). "Depression as an antecedent to heart disease among women and men in the NHANES I study." Arch Intern Med **160**: 1260-1268.

Ford DE, M. L., Chang PP, Cooper-Patrick L, Wang N-Y, Klag MJ. (1998). "Depression is a risk factor for coronary artery disease in men: the Precursors Study." Arch Intern Med **158**: 1422-1426.

Gump B.B., Matthews K.A., Eberly L.E. and Chang Y. (2005). "Depressive symptoms and mortality in men: results from the Multiple Risk Factor Intrevention Trial." Stroke **36**: 98-102.

Penninx, B. W. J. H., A. T. F. Beekman, A. Honig, D. J. H. Deeg, R. A. Schoevers, J. T. M. van Eijk and W. van Tilburg (2001). "Depression and cardiac mortality: results from a community-based longitudinal study." Archives of General Psychiatry **58**: 221-227.

Penninx BWJH, G. J., Mendes de Leon CF, Pahor M, and C. M.-C. Visser M, Wallace RB. (1998). "Cardiovascular events and mortality in newly and chronically depressed persons >70 years of age." Am J Cardiol **81**: 988-994.

Pratt LA, F. D., Crum RM, Armenian HK, Gallo JJ, Eaton WE. (1996). "Depression, psychotropic medication, and risk of myocardial infarction." Circulation **94**: 3123-3129.

Wulsin, L. R., Evans J.C., Vasan R.S., Murabito J.M., Kelly-Hayes M. and Benjamin E.J. (2005). "Depressive symptoms, coronary heart disease, and overall mortality in the Framingham Heart Study." Psychosom Med **67**: 697-702.
